# Region-Specific Neuroprotective Features of Astrocytes against Oxidative Stress Induced by 6-Hydroxydopamine

**DOI:** 10.3390/ijms20030598

**Published:** 2019-01-30

**Authors:** Masato Asanuma, Nao Okumura-Torigoe, Ikuko Miyazaki, Shinki Murakami, Yoshihisa Kitamura, Toshiaki Sendo

**Affiliations:** 1 Department of Medical Neurobiology, Okayama University Graduate School of Medical, Dental and Pharmaceutical Sciences, Okayama 700-8558, Japan; miyazaki@cc.okayama-u.ac.jp (I.M.); gmd422014@s.okayama-u.ac.jp (S.M.); 2 Department of Clinical Pharmacy, Okayama University Graduate School of Medical, Dental and Pharmaceutical Sciences, Okayama 700-8558, Japan; ph20124@s.okayama-u.ac.jp (N.O.-T.); kitamu-y@cc.okayama-u.ac.jp (Y.K.); sendou@md.okayama-u.ac.jp (T.S.)

**Keywords:** astrocyte, neuroprotection, region-specificity, striatum, mesencephalon, oxidative stress, 6-hydroxydopamine, Nrf2, phase II detoxifying molecules

## Abstract

In previous studies, we found regional differences in the induction of antioxidative molecules in astrocytes against oxidative stress, postulating that region-specific features of astrocytes lead region-specific vulnerability of neurons. We examined region-specific astrocytic features against dopaminergic neurotoxin 6-hydroxydopamine (6-OHDA) as an oxidative stress using co-culture of mesencephalic neurons and mesencephalic or striatal astrocytes in the present study. The 6-OHDA-induced reduction of mesencephalic dopamine neurons was inhibited by co-culturing with astrocytes. The co-culture of midbrain neurons with striatal astrocytes was more resistant to 6-OHDA than that with mesencephalic astrocytes. Furthermore, glia conditioned medium from 6-OHDA-treated striatal astrocytes showed a greater protective effect on the 6-OHDA-induced neurotoxicity and oxidative stress than that from mesencephalic astrocytes. The cDNA microarray analysis showed that the number of altered genes in both mesencephalic and striatal astrocytes was fewer than that changed in either astrocyte. The 6-OHDA treatment, apparently up-regulated expressions of Nrf2 and some anti-oxidative or Nrf2-regulating phase II, III detoxifying molecules related to glutathione synthesis and export in the striatal astrocytes but not mesencephalic astrocytes. There is a profound regional difference of gene expression in astrocytes induced by 6-OHDA. These results suggest that protective features of astrocytes against oxidative stress are more prominent in striatal astrocytes, possibly by secreting humoral factors in striatal astrocytes.

## 1. Introduction

It is well known that astrocytes are abundant glial cells and play an important role in the maintenance of the neuronal homeostasis, such as regulating cerebral blood flow and maintaining synaptic function. Astrocytes uptake glutamate to prevent its neurotoxicity, release several neurotrophic factors and various neurotransmitters (so-called gliotransmitters), and are the major cells to have an antioxidative defense system. A number of reports demonstrated astroglial dysfunction and reduction of astroglial antioxidative molecules in pathogenesis of various neurological disorders, such as Parkinson’s disease (PD), amyotrophic lateral sclerosis (ALS), and brain ischemia, and in their animal models [1,2]. Among various functions of astrocytes, we have been focusing on their antioxidative defense system, especially on glutathione (GSH) and metallothionein (MT) in astrocytes as a target of neuroprotection [3]. A tripeptide GSH comprising glutamate, cysteine, and glycine is the major intrinsic antioxidant against reactive oxygen species (ROS) in the brain, and is synthesized by γ-glutamyl cysteine ligase (GCL) and GSH synthetase. Since the substrate cysteine is easily auto-oxidized to cystine and neurons lack the cystine transport system, the neighboring astrocytes provide cysteine to neurons by up-taking cystine via cystine/glutamate exchange transporter (xCT, Slc7a11), synthesizing, and releasing GSH [4,5,6]. The released GSH is converted to cysteine and then up-taken into neurons as the substrate for GSH synthesis. A metal-binding protein MT is another strong antioxidative molecule, which is a low-molecular weight, cysteine-rich, metal-binding protein for zinc, copper, and cadmium to function in metal homeostasis and detoxification. The two major isoforms, MT-1 and -2, are expressed in most organs, including the brain, and are mainly produced in astrocytes [7,8]. MT shows antioxidative properties, such as free radical scavenging or quinone-quenching properties by its abundant 20-cysteine residues present in each molecule. The gene expression of GSH-related enzymes and MT is regulated by nuclear factor erythroid 2-related factor 2 (Nrf2) that is mainly expressed in astrocytes. Nrf2, inactivated by its suppressor protein ubiquitin ligase Keap1 in the normal condition, is activated by conformational change and detachment of Keap1 in response to ROS or electrophiles, and translocated into the nucleus binding to antioxidant responsive element (ARE) to promote gene expression of various phase II detoxifying and antioxidative molecules, such as GSH-related enzymes (GCL, GSH *S*-transferase (GST), GSH peroxidase (Gpx)), quinone oxidoreductase (NQO-1), superoxide dismutase (SOD), catalase, hemeoxigenase-1 (HO-1), xCT, and MT [3,9,10,11]. Both GSH and MT can prevent dopamine (DA) quinone-induced neurotoxicity as a dopaminergic neuron-specific oxidative stress by competitively binding to DA quinones [12,13,14]. Therefore, antioxidative defense systems in the astrocytes would be a potential target of the dopaminergic neuroprotection.

We previously found that zonisamide and levetiracetam markedly increased levels of anti-oxidant GSH in the basal ganglia by enhancing astroglial xCT and/or astroglial proliferation, and acts as a neuroprotectant against oxidative stress and dopaminergic neurodegeneration in a PD model [15,16]. Furthermore, the induction and secretion of strong antioxidants GSH and MT specifically in/from reactive astrocytes protect DA neurons from oxidative stress [10]. We also showed that a serotonin 5-HT1A receptor full agonist 8-OH DPAT promotes astrocyte proliferation via S100β secretion and activates the Nrf2-ARE pathway to produce MT in astrocytes by acting on astroglial 5-HT1A receptors, and consequently to prevent dopaminergic neurodegeneration in neuron-astrocyte co-cultures and in PD animal models [17]. Some phytochemicals exert protective effects on DA neurons against oxidative stress via activating Nrf2 and increasing GSH or MT in astrocytes [18,19]. These suggest that modulation of neuron-astrocyte interaction through astrocyte proliferation and up-regulation of molecules in astrocytes could be a target for development of neuroprotective drugs or techniques.

Astrocytes are morphologically classified into protoplasmic type or fibrous type. Recently, Dawson and his colleagues proposed new classification of reactive astrocytes into neurotoxic A1 astrocytes, which are induced by activated microglia, inflammatory cytokines and classical complement component, and neuroprotective A2 astrocytes [20,21], although they used only astrocytes from cortices or whole brains. In the previous studies, we found that a dramatic induction of MT after oxidative stress exposure was seen in the striatal astrocytes but not in cerebro-cortical astrocytes [10]. Furthermore, when the dopaminergic neurons are damaged, uptake of DA into astrocytes was increased in the striatum [22] but not in the midbrain. Thus, it is postulated that there are regional differences in the response of astrocytes and the induction of antioxidative molecules in astrocytes against oxidative stress, and that region-specific features of astrocytes lead to region-specific vulnerability of neurons against a certain stimulus. Therefore, we examined region-specific astrocytic features against dopaminergic neurotoxin 6-hydroxydopamine (6-OHDA) as an oxidative stress using a co-culture of mesencephalic neurons and striatal or mesencephalic astrocytes, in the present study.

## 2. Results

### 2.1. Regional Difference in Astroglial Neuroprotective Effects

**Figure 1:**
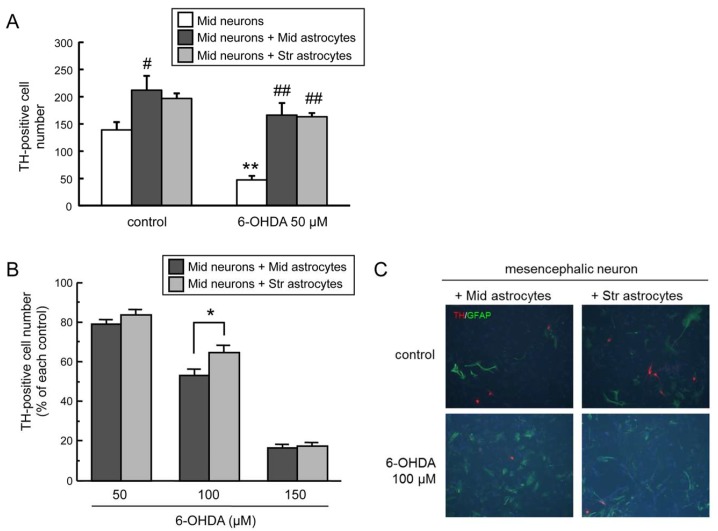
(**A**) Neuroprotective effects of co-culture with mesencephalic or striatal astrocytes against 6-hydroxydopamine (6-OHDA)-induced toxicity in mesencephalic neurons. Mesencephalic neurons were exposed to 6-OHDA (50 µM) for 24 h co-existing with mesencephalic or striatal astrocytes. Each value is mean of the number of tyrosine hydroxylase (TH)-positive neurons ± SEM (*n* = 4); ** *p* < 0.01 vs. each control group, # *p* < 0.05; ## *p* < 0.01 vs. each neuron group. (**B**) Regional difference in astroglial neuroprotective effect. Cell viability of TH-positive DA neurons co-cultured with mesencephalic or striatal astrocytes treated with 6-OHDA (50–150 µM) for 24 h. Data are means ± SEM (*n* = 4) expressed as percentage of each control group; * *p* < 0.05 between indicated two groups. (**C**) Representative fluorescence photomicrographs of TH (red) and glial fibrillary acidic protein (GFAP) (green) double immunostaining of mesencephalic neurons co-cultured with mesencephalic or striatal astrocytes treated with 6-OHDA (100 µM) for 24 h. Images are taken at 200× magnification.

Previous studies indicated that mesencephalic astrocytes promote the survival of mesencephalic neuronal cells against 6-OHDA toxicity in the neuron-astrocyte co-culture system [23]. To confirm the neuroprotective effect of astrocytes in the mesencephalon and striatum against 6-OHDA-induced neurotoxicity, we treated enriched mesencephalic neuronal cultures, mesencephalic neuron-mesencephalic astrocyte co-cultures, and mesencephalic neuron-striatal astrocyte co-cultures with 6-OHDA (50 µM) for 24 h. The decrease in tyrosine hydroxylase (TH)-positive dopaminergic neurons induced by 6-OHDA was inhibited in the mesencephalic neurons co-cultured with astrocytes from both brain regions (Figure 1A).

Next, to elucidate regional difference in the neuroprotective effect of astrocytes, both co-cultures were exposed to 6-OHDA (50–150 µM) for 24 h. Regional differences of astrocytes in neuronal survival were seen at the dose of 100 µM in the 6-OHDA treatment. Survival of TH-positive DA neurons co-cultured with striatal astrocytes after 6-OHDA (100 µM) exposure was significantly higher than that with mesencephalic astrocytes (Figure 1B). However, there were no apparent morphological differences between mesencephalic and striatal astrocytes in the double immunohistochemistry of TH and reactive astrocytic marker glial fibrillary acidic protein (GFAP) in the mesencephalic neurons co-cultured with mesencephalic or striatal astrocytes exposed to 100 µM 6-OHDA for 24 h (Figure 1C).

### 2.2. Regional Difference in Glia Conditioned Medium (GCM)

**Figure 2:**
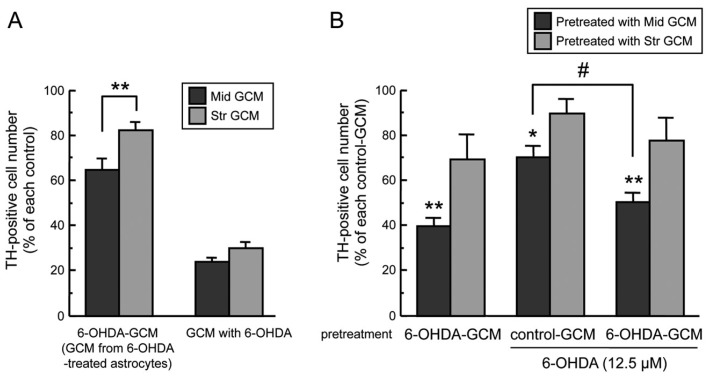
(**A**) Regional difference of glia conditioned media (GCM). Cell viability of TH-positive dopaminergic neurons co-incubated with mesencephalic or striatal GCM (control-GCM, 6-OHDA-GCM, GCM with 6-OHDA (100 µM) for 24 h. Each value is mean ± SEM (*n* = 4) expressed as percentage of each control-GCM group; ** *p* < 0.01 between indicated two groups. (**B**) Neuroprotective effect of striatal GCM. Mesencephalic neurons were pre-treated with control-GCM or 6-OHDA-GCM for 24 h, replaced with fresh medium, and then treated with 6-OHDA (12.5 µM) for another 24 h. Data are means ± SEM (*n* = 3–4) expressed as percentage of TH-positive cell number of vehicle-treated group after each control-GCM pretreatment; * *p* < 0.05, ** *p* < 0.01 vs. each control-GCM groups, # *p* < 0.05 between indicated two groups.

Glia conditioned media (GCM) from glial cells promotes the survival of neuronal cells [24,25,26,27] and astrocytes release GSH into culture medium [28]. Furthermore, astroglial neuroprotective effects in the co-culture system were different between mesencephalic astrocytes and striatal astrocytes (Figure 1). Such regional differences might be based on humoral factors secreted from astrocytes. Therefore, we examined neuroprotective effects of GCM from mesencephalic and striatal astrocytes against 6-OHDA-induced neurotoxicity in mesencephalic DA neurons. The viability of TH-positive midbrain neurons by 24-h incubation with GCM from 6-OHDA-treated astrocytes (6-OHDA-GCM) was higher compared to that incubated with control GCM plus 6-OHDA (100 µM) (Figure 2A, 6-OHDA-GCM vs. GCM with 6-OHDA). When the mesencephalic neuronal culture was incubated in GCM from mesencephalic astrocytes (Mid GCM) or striatal astrocytes (Str GCM) treated with 6-OHDA (100 µM) for 24 h, the GCM of 6-OHDA-treated striatal astrocytes showed a greater neuroprotective effect on the viability of TH-positive neurons than that from mesencephalic astrocytes (Figure 2A, 6-OHDA-GCM).

We then assessed neuroprotective effects of pretreatment with GCM against 6-OHDA neurotoxicity. The mesencephalic neuronal culture was pre-incubated in control-GCM or 6-OHDA-GCM for 24 h. After the pretreatment with each GCM, the medium was changed to fresh normal medium, and mesencephalic neurons were treated with 6-OHDA (12.5 µM) for another 24 h. The number of mesencephalic TH-positive dopaminergic neurons was decreased to approximate 70% of control after treatment with 6-OHDA (12.5 µM) alone. The decrease in dopaminergic neurons induced by 6-OHDA was inhibited by the pre-treatment of control GCM and 6-OHDA-GCM from striatal astrocytes, but not by the pre-incubation with either GCM from mesencephalic astrocytes (Figure 2B). Furthermore, only pre-treatment with GCM from 6-OHDA-treated mesencephalic astrocytes (Mid-6-OHDA-GCM) showed a dramatic reduction of TH-positive dopaminergic neurons without any additional 6-OHDA treatment, but pre-treatment with GCM from 6-OHDA-treated striatal astrocytes (Str-6-OHDA-GCM) did not.

### 2.3. Altering Expression of Genes Induced by 6-OHDA in Astrocytes

**Figure 3:**
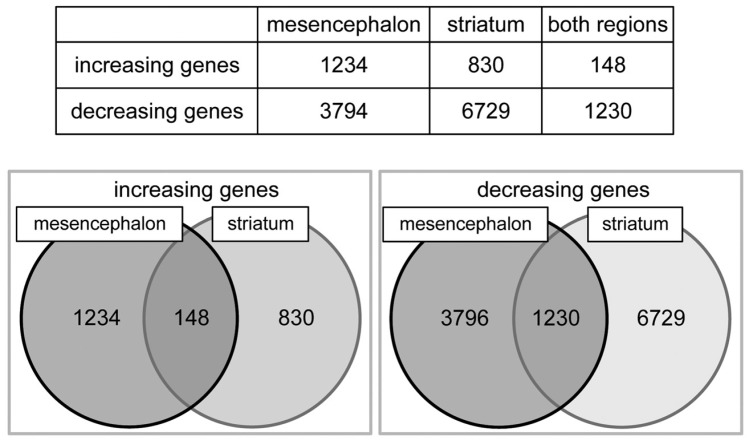
Alterations of mRNA gene expression in mesencephalic or striatal astrocytes treated with 6-OHDA. Cultured mesencephalic or striatal astrocytes were treated with 6-OHDA (100 µM) for 24 h. The mRNA gene expression ratio was expressed in fluorescence intensity of 6-OHDA-treated group/fluorescence intensity of control group on a cDNA microarray. The increasing gene was defined as Log_2_ (ratio) >1 if ratio was more than double, and the decreasing gene was defined as Log_2_ (ratio) <−1 if ratio was less than one-half.

In order to clarify gene expression comprehensively, we conducted a cDNA microarray (SurePrint G3 Rat Gene Expression 8 × 60K; Agilent Technology), on which cDNA probes of 19,742 genes were blotted, and we detected increasing or decreasing mRNA expression in mesencephalic or striatal astrocytes by the 6-OHDA treatment (Figure 3). In mesencephalic astrocytes, 1234 mRNAs were increased, and 3794 genes decreased by the treatment of 6-OHDA (100 µM) for 24 h. In striatal astrocytes, expression of 830 mRNAs was increased, and that of 6729 genes decreased. Among these genes, expression of 148 mRNAs was up-regulated, whereas 1230 genes were down-regulated in both mesencephalic and striatal astrocytes after 24-h 6-OHDA treatment. Thus, the number of altered genes in both mesencephalic and striatal astrocytes was quite fewer than that we expected. There is a profound regional difference in gene expression in astrocytes induced by 6-OHDA.

In addition, we searched for altering genes that were involved in antioxidative defense system or detoxifying system, because the pathway analysis of cDNA microarray showed differential gene expression of phase II, III detoxifying molecules in the striatal astrocytes by the 6-OHDA treatment. The mRNA expression of glutathione *S*-transferase (GST), glutathione peroxidase (Gpx), and quinone oxidoreductase (NQO-1) were increased in mesencephalic and striatal astrocytes treated with 6-OHDA (Table 1). Furthermore, genes in which expression was increased only in striatal astrocytes by 6-OHDA treatment were hemeoxigenase-1 (HO-1), catalase, glucose-6-phosphate dehydrogenase (G6PD), xCT, p-glycoprotein (P-gp, MDR1, Abcb1), and multidrug resistance-associated protein 4 (MRP4, Abcc4). Comparing gene expressions in mesencephalic and striatal astrocytes, the increasing ratio of these gene expressions in striatal astrocytes was much greater than that in mesencephalic astrocytes, except Gpx. These resulting data in the cDNA microarray suggest that the gene expression involved in antioxidative and detoxifying functions was up-regulated in striatal astrocytes, compared with midbrain astrocytes, by the treatment of 6-OHDA.

### 2.4. GSH Levels in 6-OHDA-Treated Astrocytes

**Figure 4:**
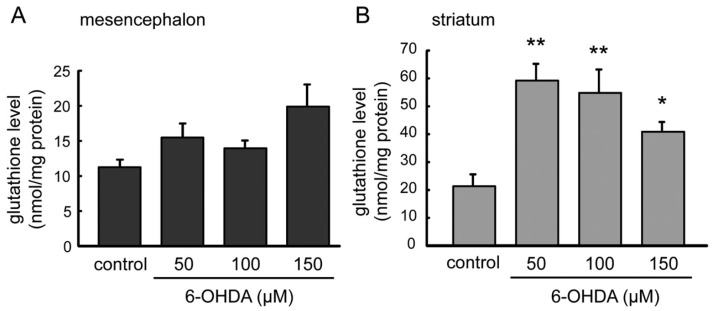
Total glutathione levels in astrocytes treated with 6-OHDA. Mesencephalic (**A**) or striatal (**B**) astrocytes were treated with 6-OHDA (50–150 µM) for 24 h. Each value is presented as mean ± SEM (*n* = 5–6); * *p* < 0.05, ** *p* < 0.01 vs. control group.

GSH was known for mainly as an anti-oxidative factor in brain. GSH is an effective antioxidative by clearing not only ROS but also DA quinone, which were produced from excess DA. In the cDNA microarray analysis, mRNA expression of GSH-related molecules, such as GST, xCT, MRP4, were up-regulated in the striatal astrocytes (Table 1). We measured GSH levels in mesencephalic and striatal astrocytes 24 h after the treatment with 6-OHDA (50–150 µM) (Figure 4). Although significant change induced by 6-OHDA treatment was not detected in GSH contents in mesencephalic astrocyte (Figure 4A), total GSH content increased significantly in 6-OHDA treatment (50–150 µM) in striatal astrocytes (Figure 4B). In particular, total GSH content in striatal astrocytes was markedly increased by 50 µM 6-OHDA treatment, and was gradually decreased in inverse proportion to the 6-OHDA concentration.

### 2.5. Expression of Nrf2 and Its Regulating Phase-II Detoxifying Molecules in 6-OHDA-Treated Astrocytes

**Figure 5:**
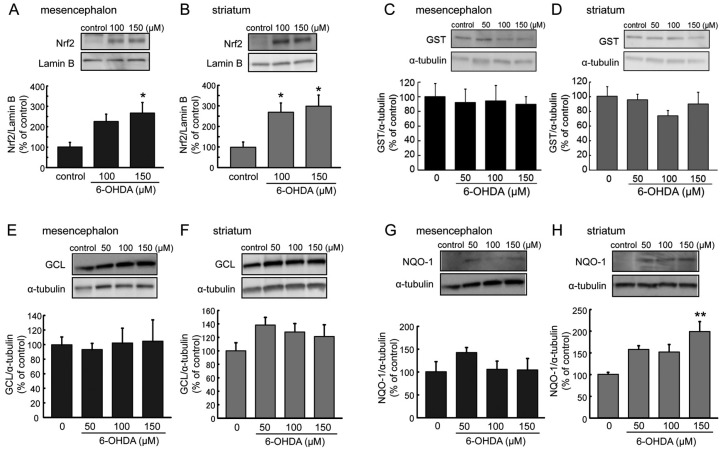
(**A**,**B**) Effects of 6-OHDA treatment (100–150 µM) for 6 h on nuclear Nrf2 expression in mesencephalic (**A**) or striatal (**B**) astrocytes. Data are means ± SEM (*n* = 5–6); * *p* < 0.05 vs. control group. (**C**–**H**) Expression of glutathione *S*-transferase (GST) (**C**,**D**), γ-glutamyl cysteine ligase (GCL) (**E**,**F**), and quinone oxidoreductase (NQO-1) (**G**,**H**) protein in mesencephalic (**C**,**E**,**G**) or striatal (**D**,**F**,**H**) astrocytes after the treatment with 6-OHDA (50–150 µM) for 24 h. Data are means ± SEM (*n* = 4–6). ** *p* < 0.01 vs. control group.

The gene expression of the phase II detoxifying molecules was regulated by antioxidative master transcription factor Nrf2. The results of alteration of gene mRNA expression in the cDNA microarray (Table 1) suggested a possibility of induction of phase II detoxifying and antioxidative molecules regulated by Nrf2. To investigate activation of Nrf2 expression and phase II detoxifying and antioxidative proteins in 6-OHDA-induced toxicity, we examined the expression level of activated Nrf2 in the nuclear fraction (Figure 5A,B) and protein levels of GST, GCL, and NQO-1 (Figure 5C–H) in total cell lysate in mesencephalic or striatal astrocyte treated with 6-OHDA for 24 h, by Western blot analysis. 

The treatment with 6-OHDA (100–150 µM) for 6 h increased nuclear Nrf2 expression in both mesencephalic and striatal astrocytes, and a significant nuclear activation of Nrf2, was seen even at the dose of 100 µM 6-OHDA in striatal astrocytes (Figure 5A,B). In contrast to the gene expression analysis by cDNA microarray, protein levels of GST were not changed by 6-OHDA treatment for 24 h in either mesencephalic and striatal astrocytes (Figure 5C,D). In the striatal astrocytes, the 24-h 6-OHDA treatment showed increasing tendency, but not significantly, in protein levels of GCL, which is not included in the cDNA microarray (Figure 5E,F). Expression level of NQO-1 protein was increased by the treatment with 6-OHDA in striatal astrocytes but not in mesencephalic astrocytes (Figure 5G,H). The NQO-1 protein was markedly and significantly up-regulated by 6-OHDA (150 µM) for 24 h in striatal astrocytes.

### 2.6. Induction of Oxidative Stress in 6-OHDA-Treated Astrocytes

**Figure 6:**
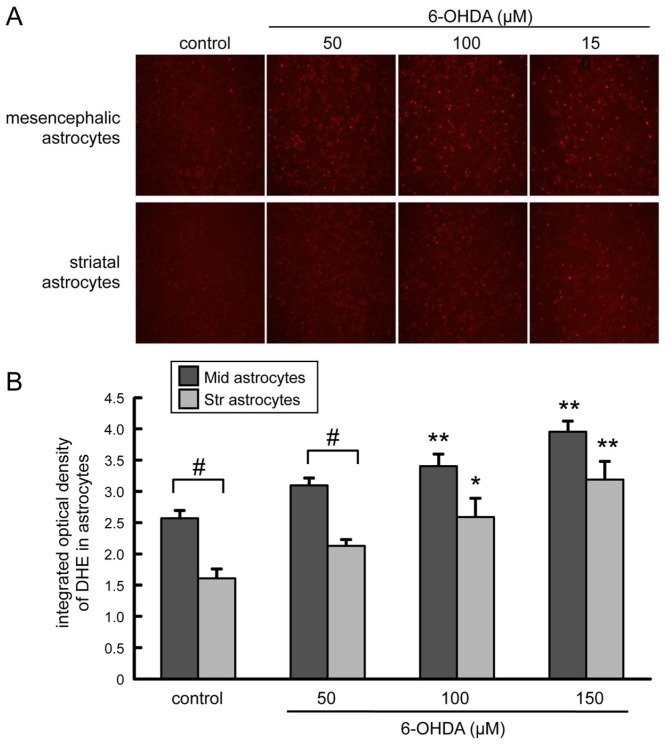
(**A**) Representative fluorescence photomicrographs of dihydroethidium (DHE)-derived fluorescence (red) on cultured mesencephalic or striatal astrocytes treated with 6-OHDA (50–150 µM) for 24 h. Images are taken at 100× magnification. (**B**) Effect of 6-OHDA treatment on superoxide production in astrocytes. Signal intensity of DHE-positive astrocytes (mesencephalic or striatal astrocytes) treated with 6-OHDA (50–150 µM) for 24 h. Data are means ± SEM (*n* = 6); * *p* < 0.05; ** *p* < 0.01 vs. each control group, # *p* < 0.05 between indicated two groups.

To study a possible regional difference in the induction of oxidative stress related to the neuroprotective effect of astrocytes, we examined production of ROS in mesencephalic or striatal astrocytes treated with 6-OHDA (Figure 6). Red fluorescence signal of superoxide indicator dihydroethidium (DHE) was significantly increased in both mesencephalic and striatal astrocytes by 6-OHDA (100, 150 µM) treatment in a dose-dependent manner. The DHE signal was much higher in mesencephalic astrocytes than that in striatal astrocytes at any of the doses of 6-OHDA examined (50, 100, 150 µM), even in the control group.

### 2.7. Quinone Formation in 6-OHDA-Treated Astrocytes or Neuron-Astrocyte Co-Cultures

**Figure 7:**
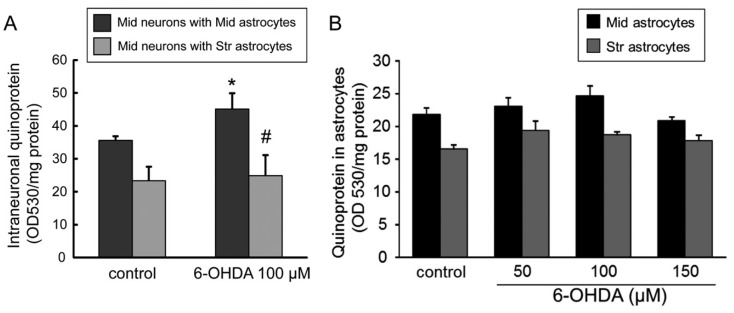
(**A**) Changes in quinoprotein formation in mesencephalic neurons co-cultured with mesencephalic or striatal astrocytes treated with 6-OHDA (100 µM) for 24 h. Each value is mean ± SEM (*n* = 4); * *p* < 0.05 vs. each control group, # *p* < 0.05 vs. midbrain neurons with midbrain astrocytes. (**B**) Effect of 6-OHDA treatment (50–150 µM) for 24 h on quinoprotein formation in mesencephalic or striatal astrocytes. Data are means ± SEM (*n* = 5–6).

Furthermore, we examined quinone formation in astrocytes or neuron-astrocyte separate co-cultures treated with 6-OHDA. The level of quinoprotein protein-bound quinone in mesencephalic neurons was significantly increased by 6-OHDA treatment (100 µM) for 24 h when neurons were separately co-cultured with mesencephalic astrocytes, but not with striatal astrocytes (Figure 7A). Quinoprotein level in striatal astrocytes was lower than that in mesencephalic astrocytes. However, the 6-OHDA treatment (50–150 µM) for 24 h did not alter quinoprotein in both mesencephalic and striatal astrocytes (Figure 7B).

## 3. Discussion

Oxidative stress is a common pathogenesis in neurodegenerative diseases. The region-specific vulnerability of neurons against oxidative stress has been thought to cause region-specific neurodegeneration. As mentioned above, we previously found regional differences in the induction of antioxidative molecules in astrocytes against oxidative stress in dopaminergic neurodegeneration in the model of Parkinson′s disease, and suggested a possibility that region-specific features of astrocytes lead region-specific vulnerability of neurons. In the present study, we examined region-specific astrocytic features against dopaminergic neurotoxin 6-hydroxydopamine (6-OHDA) as dopaminergic oxidative stress using co-cultures of mesencephalic neurons and mesencephalic or striatal astrocytes. Co-culturing with astrocytes protected mesencephalic DA neurons from 6-OHDA-induced toxicity, coinciding with the protective effects of astrocytes against neurotoxicity induced by hydrogen peroxide [29] and 6-OHDA [23]. Survival of DA neurons co-cultured with striatal astrocytes after 6-OHDA exposure was significantly higher than that with mesencephalic astrocytes (Figure 1). Furthermore, pre-treatment of GCM from striatal astrocytes made mesencephalic DA neurons more resistant to 6-OHDA than GCM from mesencephalic astrocytes (Figure 2). We previously reported that GCM from mesencephalic astrocytes showed protective effects against 6-OHDA-induced neuronal death of mesencephalic DA neurons [23]. However, brain regional differences of the protective property of astrocytes have been obscure. The present resulting data showed regional differences of neuroprotective property between striatal and mesencephalic astrocytes against oxidative stress-induced dopaminergic neurotoxicity, suggesting that the release of humoral neuroprotective molecules from striatal astrocytes is much greater than that from mesencephalic astrocytes. Although the viability of DA neurons with addition of GCM from 6-OHDA-treated astrocytes (6-OHDA-GCM) was lower than that with incubation of control GCM, it was much higher than that in the case of direct addition of 6-OHDA to GCM (GCM with 6-OHDA) (Figure 2A). Therefore, neuroprotective effects of released factors from striatal astrocytes might overwhelm toxic effects of remaining 6-OHDA in GCM. The pretreatment with GCM from 6-OHDA-treated mesencephalic astrocytes, but not GCM from striatal astrocytes, declined the number of mesencephalic DA neurons with or without additional 6-OHDA treatment (12.5 µM), and it did not aggravate toxic effects of 6-OHDA, which declined DA neurons to 70% of control (Figure 2B). This implies another region-specific property of astrocytes exposed to oxidative stress that mesencephalic astrocytes release neurotoxic molecules, which showed more greater toxicity than that of 6-OHDA.

The comprehensive analysis using a cDNA microarray showed a unique altering pattern of mRNA gene expression in 6-OHDA-treated mesencephalic or striatal astrocytes (Figure 3). Among 19,742 genes blotted, a relatively small number of gene expression was up-regulated (148 genes) or down-regulated (1230 genes) in both mesencephalic and striatal astrocytes by 6-OHDA treatment. In other words, we could clarify apparent regional differences in 6-OHDA-induced alteration of mRNA expression in astrocytes. Furthermore, the pathway analysis of cDNA microarray indicated that genes in which expression was increased dominantly or only in striatal astrocytes by 6-OHDA were anti-oxidative or phase II detoxifying molecules, such as GST, NQO-1, HO-1, catalase, G6PD, xCT, and phase III detoxifying molecules MDR1 (P-gp) and MRP4 (Table 1). All of these mRNA gene expressions were regulated by antioxidants and detoxification master transcription factor Nrf2 [3,6,9,11,30,31,32,33]. We confirmed marked increases in the levels of nuclear Nrf2, GSH, and NQO-1 protein and an increasing tendency of GCL specifically in striatal astrocytes after 6-OHDA treatment (Figure 4 and Figure 5). GSH can scavenge not only ROS but also DA quinones and NQO-1 reduces quinones to ameliorate quinone toxicity [12,13]. The xCT on astrocytes up-takes cystine to promote GSH synthesis and its release, supplying the substrate cysteine to neighboring neurons, as mentioned above [4,5,6]. MRP4 is one of phase III detoxifying molecules, which is involved in efflux of drugs and toxins, and plays an important role of export GSH or GSH-conjugates [34,35]. Although MDR1 (P-gp) is not involved in export of GSH-conjugates [36], it may act in the efflux and metabolism of the toxin 6-OHDA. We previously reported that excess DA is taken up by striatal astrocytes via DA transporter activates Nrf2 and up-regulates GSH synthesis and MT expression to protect neurons from free radical and/or quinone-induced toxicity [10]. As well as DA, 6-OHDA is also up-taken through DA transporter to cause neurotoxicity via producing ROS and quinones by auto-oxidation [37,38]. Levels of superoxide and quinoprotein in striatal astrocytes were much lower than those in mesencephalic astrocytes at normal state and any dose of 6-OHDA (Figure 6 and Figure 7). Thus, Nrf2-regulating anti-oxidative or phase II, III detoxifying molecules, which are involved in GSH synthesis/export (xCT, GCL, GSH, MRP4) and quinone reduction (NQO-1), were up-regulated specifically in striatal astrocytes against dopaminergic neurotoxin 6-OHDA. Such a high responsiveness of these molecules in striatal astrocytes could diminish ROS and/or quinone-induced neurotoxicity.

A number of studies indicated involvement of Nrf2-regulating anti-oxidative or detoxifying molecules in astrocytes in pathogenesis and neuroprotective strategies of PD [3]. Knock-out of parkin is a mutation which causes familial PD produced astroglial dysfunction and age-dependent vulnerability of neurons against oxidative stress that was corrected by supplementation with GSH [39]. DJ-1, a causative gene of a familial PD, is dominantly expressed in astrocytes to stabilize Nrf2 [40], and its knock-down in astrocytes impaired mitochondrial function and neuroprotective effects of astrocytes against dopaminergic neurotoxin [41,42]. NQO-1 expression increased in both astrocytes and neurons in the substantia nigra pars compacta of PD patients [43]. Astrocyte-specific overexpression of Nrf2 almost completely protected DA neurons from neurotoxin MPTP-induced neurotoxicity [44] and dramatically reduced α-synuclein aggregation and gliosis in mutant α-synuclein PD model [45]. Furthermore, we previously reported that zonisamide and levetiracetam enhanced astroglial xCT expression and/or astroglial proliferation, increased GSH, and protected dopaminergic neurodegeneration in PD model [15,16], and that 5-HT1A receptor agonist 8-OH DPAT promoted Nrf2 activation, S100β, and MT secretion in striatal astrocytes to protect DA neurons in culture and in an animal model of PD [17]. Therefore, highly responsive and neuroprotective features of antioxidative defense systems in striatal astrocytes against oxidative stress shown in the present study could be a potential target to develop disease-modifying strategies for PD.

Striatal astrocytes surrounding nerve endings of DA neurons are constitutively exposed to DA-derived oxidative stress. This specific feature of the striatal astrocyte might lead to its high responsiveness to the antioxidative defense system. Although the mechanism of such regional differences in neuroprotective features between mesencephalic and striatal astrocytes are obscure, the present study indicates that prominent release of humoral neuroprotective factors, such as GSH, or reduced release of neurotoxic molecules from striatal astrocytes, may contribute to the region-specific neuroprotective properties of striatal astrocytes against oxidative stress. On the other hand, the lower responsiveness of mesencephalic astrocytes against oxidative stress might cause the region-specific vulnerability of neurons in the midbrain.

## 4. Materials and Methods

### 4.1. Reagents and Animals

Dulbecco’s modified Eagle’s medium and TRIzol Reagent were purchased from Invitrogen. (San Diego, CA, USA). 6-OHDA was from Sigma-Aldrich (St. Louis, MO, USA). The cDNA micro array kits were from Agilent (Agilent Technologies, Santa Clara, CA, USA). Pregnant Sprague-Dawley rats were purchased from Charles River Japan, Inc. (Yokohama, Japan). All animal procedures were in strict accordance with the Guideline for Animal Experiments of Okayama University Advanced Science Research Center, and approved by the Animal Care and Use Committee of Okayama University Advanced Science Research Center (OKU-2014042 and OKU2017132, approved on 1 April 2014 and 6 June 2017, respectively).

### 4.2. Cell Cultures

Primary cultured neurons were prepared from the mesencephalon and astrocytes were from the mesencephalon and striata of Sprague-Dawley rat embryos at 15 days of gestation using the method described previously [17]. The tissues were incubated for 15 min in 0.125% trypsin at 37 °C and then centrifuged (1500 *g*, 5 min). The cell pellet was treated with 0.004% DNaseI for 8 min at 37 °C and then recentrifuged (1500 *g*, 5 min). The cell pellet was then resuspended in a small volume of Dulbecco’s modified Eagle’s medium (DMEM, Waltham, MA, USA) containing 10% fetal bovine serum (FBS).

The mesencephalic or striatal cell for astrocytic culture was plated in DMEM containing 10% FBS at a density of 2 × 10^5^ cells/cm^2^ in 6-well plates coated with poly-d-lysine (Becton Dickinson, Franklin Lakes, NJ, USA). Within 24 h after initial plating, the medium was replaced with fresh medium. The cells were cultured for 4–13 days in same medium, and then subcultured to obtain enriched astrocyte cultures. Mesencephalic or striatal astrocytes were plated at density of 3.6 × 10^4^ cells/cm^2^ onto 6-well culture plates (Becton Dickinson) for extraction of total RNA and total protein or nuclear protein, or 96-well culture plates (Becton Dickinson) for dihydroethidium staining. It is confirmed that over 95% of these cultured glial cells showed GFAP immunoreactivity.

The mesencephalic cells for neuronal culture were plated in the same medium at a density of 2×10^5^ cells/cm^2^ in four-chamber culture slides coated with poly-D-lysine (Becton Dickinson) for immunohistochemical analysis. Within 24 h after initial plating, the medium was replaced with fresh medium supplemented with 2 µM of cytosine-β-d-arabinofuranoside (Ara-C) to inhibit the replication of non-neuronal cells, and incubated for a further 5 days. In neuron-rich cultures, 95% of the cells were immunoreactive for the neuronal marker microtubule-associated protein 2 (MAP2).

For neuron-astrocytes of mesencephalon or striatum co-cultures for immunohistochemical analysis, each group of astrocytes were seeded at a density of 4 × 10^4^ cells/cm^2^ directly onto mesencephalic neuronal cell layers, which were already cultured in 4-chamber culture slides for 4 days as described. In the same way, for neuron-astrocytes of the co-cultures for measurement of intraneuronal levels of quinoprotein protein-bound quinone, the astrocytes were seeded on the insert over mesencephalic neuronal cell layers. The cells were then co-cultured for another 2 days. All cultures were maintained at 37 °C in a 5% CO_2_/95% air mixture.

### 4.3. Preparation of Conditioned Medium

The mesencephalic or striatal astrocytes were plated onto 6-well plates (Becton Dickinson) and grown in DMEM containing 10% FBS for 13 days. To prepare each astrocyte conditioned medium, each group of astrocytes was treated with 100 µM 6-OHDA (mesencephalic-6-OHDA-GCM, striatal-6-OHDA-GCM) or vehicle (mesencephalic-GCM, striatal-GCM) for 24 h. Then, each GCM and 6-OHDA-GCM were collected, centrifuged at 1500 rpm for 3 min to remove cellular debris, and the supernatants stored at −80 °C until use.

### 4.4. Cell Treatments

Cultured mesencephalic or striatal astrocytes were exposed to 100 µM 6-OHDA for 24 h for extraction of total RNA, and were exposed to 6-OHDA (50–150 µM) for 24 or 6 h for total or nuclear protein. Neuron-rich cultures and neuron-mesencephalic or striatal astrocyte co-cultures were treated with 6-OHDA (50–150 µM) for 24 h. To examine the regional differences of GCM, mesencephalic neuronal cultures were cultured with GCM from mesencephalic or striatal astrocyte treated 6-OHDA (100 µM) or vehicle (mesencephalic-6-OHDA-GCM, striatal-6-OHDA-GCM, mesencephalic-GCM, striatal-GCM) for 24 h. To examine the mechanism and regional differences of astrocytic neuroprotection against 6-OHDA-related neurotoxicity, mesencephalic neuronal cultures were treated with GCM from mesencephalic or striatal astrocyte treated 6-OHDA or vehicle (mesencephalic-6-OHDA-GCM, striatal-6-OHDA-GCM, mesencephalic-GCM, striatal-GCM) for 24 h. After 24 h treatment with each GCM, culture medium was changed to fresh normal medium, and then neurons were exposed to 6-OHDA (12.5 µM) for 24 h. All astrocyte-conditioned media contained 2 µM Ara-C throughout the culture period to inhibit the replication of non-neuronal cells.

### 4.5. Immunohistochemistry

The cells were fixed with 4% paraformaldehyde (PFA) for 30 min at RT and washed in 10 mM phosphate-buffered saline (PBS, pH 7.4) After blocking with 2.5% normal goat serum for 20 min at RT, the cells were reacted with primary antibodies overnight at 4 °C; mouse anti-TH (1:1000; Millipore, MA, USA) with rabbit anti-GFAP (1:2000; Dako Cytomation, Glostrup, Denmark), diluted in 10 mM PBS containing 0.1% Triton X-100 (0.1% PBST). After washing in 10 mM PBS, the secondary antibodies used were goat anti-mouse IgG conjugated to Alexa Fluor 594 (1:250; Invitrogen) or goat anti-rabbit IgG conjugated to Alexa Fluor 488 (1:500; Invitrogen). Finally, the cells were counterstained with Hoechst 33342 nuclear stain before mounting with fluoromounting medium (Dako).

For analysis of the neuroprotective effects of astrocytes, TH-immunopositive cells were counted under fluorescence microscope (Olympus BX50-FLA, Tokyo, Japan) in all areas of each chamber slide. Images were taken at 200× magnification.

For detection of intracellular superoxide anion production induced by the 6-OHDA treatment, 4 images were randomly chosen in each group. The integrated density of DHE-positive signals was measured quantitatively using a Macintosh computer-based image analysis system (NIH ImageJ 1.45s, NIH, Bethesda, MD, USA).

### 4.6. cDNA Microarray

Genes induced by 6-OHDA (100 µM) in astrocytes of mesencephalon or striatum were searched comprehensively by microarray (Agilent Technologies). The cells from 6 wells in each group, mesencephalic or striatal astrocytes treated with vehicle or 100 µM 6-OHDA for 24 h (Mid-control, Mid-6-OHDA, Str-control, Str-6-OHDA), were collected and homogenized in TRIzol^®^ reagent (Invitrogen), and total RNA was extracted. Sample labeling by Cy3 and array hybridization were performed according to One-Color Microarray-Based Gene Expression Microarrays Analysis (Agilent Technology). Total RNA from each sample was linearly amplified and labeled with Cy3. Total RNA was checked for quantity by using Agilent 2100 Bioanalyze. The Cy3-labeled cRNA was fragmented and then heated at 60 °C for 30 min. Next, it was hybridized to Agilent Expression Array (SurePrint G3 Rat Gene Expression 8 × 60 K; Agilent Technology), on which cDNA probes of 19,742 genes were blotted at 65 °C for 17 h. The hybridized array was washed by Gene Expression Wash Buffer Pack (Agilent Technology), and scanned using the Agilent DNA Microarray Scanner (G2565CA). The level of gene expression was calculated from fluorescence intensity quantified using Agilent Feature Extraction software. We normalized the gene transcript expression valued for the control samples on each chip and then compared differentially expressed mRNAs between control and 6-OHDA-treated groups. Gene expression ratio was expressed in fluorescence intensity of 6-OHDA-treated group/fluorescence intensity of control group. Then, increasing genes were defined as Log_2_ (ratio) >1 if ratio was more than double, and decreasing genes were defined as Log_2_ (ratio) <−1 if ratio was less than one-half. 

### 4.7. Measurement of Total Glutathione (GSH)

Total GSH levels were determined using the enzymatic recycling method of Tietze with some modification [46]. For the preparation of the GSH sample, cell pellets were resuspended in 0.1 M phosphate buffer (PB) (pH 7.4) and homogenized with 10% trichloroacetic acid. Acid extracts were mixed with 0.01 M PB (pH 7.4), NADPH (4 mM), and GSH reductase (6 U/mL). Samples were incubated for 5 min at 37 °C. The 5,5′-dithiobis (2-nitrobenzoic acid) (10 mM) was added just before reading the absorbance. The formation of 2-nitro-5-thiobenzoic acid was measured at 412 nm for 10 min. Total GSH content was determined from a standard curve that was constructed using known amounts of GSH. 

### 4.8. Western Blot Analysis

Total cell lysates from cultured mesencephalic or striatal astrocytes treated with 6-OHDA for 24 h were extracted and prepared using the PARIS protein and RNA isolation system (Ambion, Austin, TX, USA), according to the protocol described in the kit. In the same way, nuclear lysates from cultured mesencephalic or striatal astrocytes treated with 6-OHDA for 6 h were extracted and prepared. Western blot analysis was performed as described previously [17]. On polyvinylidene difluoride membranes (Hybond P, GE Healthcare, Buckinghamshire, UK), proteins were labelled using the following antibodies: total cell lysates; rabbit polyclonal anti-GCL (= γ-glutamylcysteine synthetase) (diluted 1:200, LAB VISION, Fremont, CA, USA), rabbit polyclonal anti-GST (diluted 1:200, Santa Cruz, CA, USA), or goat polyclonal anti-NAD(P)H: NQO-1 (diluted 1:200, Santa Cruz), or mouse monoclonal anti-α-tubulin (diluted 1:200, Sigma) antibodies, nuclear lysates; rabbit polyclonal anti-Nrf2 (diluted 1:200, Santa Cruz) or goat polyclonal anti-lamin B (diluted 1:200, Santa Cruz) antibodies. After incubation with the corresponding horseradish peroxidase-conjugated secondary antibody, specific signals were visualized by chemiluminescence using the ECL western blotting detection system (GE Healthcare). Images were obtained and quantified using a FUJIFILM Luminescent Image Analyzer LAS-3000 (FUJIFILM, Tokyo, Japan) and Multi Gauge (v3.0, FUJIFILM, Tokyo, Japan) software. For quantitative analysis, the signal ratio of a specific protein (relative chemiluminescence unit) to that of constitutively expressed α-tubulin or lamin B protein were calculated to normalize for loading and transfer artifacts. 

### 4.9. Measurement of Protein-Bound Quinone (Quinoprotein)

Mesencephalic neurons co-cultured with mesencephalic or striatal astrocytes were exposed to 6-OHDA (150 µM) for 24 h. Also, mesencephalic or striatal astrocytes were exposed to 6-OHDA (50–150 µM) for 24 h. Total cell lysates were then prepared by using PARIS kit, as mentioned before. Protein-bound quinones (quinoprotein) in the lysate were detected using the NBT/glysinate assay as described previously [47], with minor modifications. Briefly, the protein sample was added to 100 µL of NBT reagent (0.24 mM NBT in 2 M potassium glysinate, pH 10) followed by incubation in the dark for 2 h on a shaker. Absorbances were measured at 550 nm.

### 4.10. Measurement of Intracellular Superoxide Anions

Intracellular superoxide anions induced by 6-OHDA were detected by using superoxide indicator DHE. Mesencephalic or striatal astrocytes plated on 96-well plates were exposed to 6-OHDA (50–150 µM) for 24 h. After 24 h treatment, the cells were washed with PBS, then added to 2 µM DHE and incubated for 10 min at 37 °C. The 96-well plates were observed by microscope (Olympus, IX-71; DP-70).

### 4.11. Protein Determination

For measurement of total glutathione, the protein contents were determined using the Bio-Rad protein assay kit, based on the method of Bradford, with bovine serum albumin as a standard. For the other assays, they were determined using the Lowry-based Bio-Rad DC protein assay kit (Bio-Rad, Richmond, CA, USA) with bovine serum albumin as a standard. 

### 4.12. Satistical Analysis

Data are presented as means ± SEM. The number of wells or chambers in each group of the experiments was indicated in the figure legend. Differences between groups were examined for statistical significance using one-way analysis of variance (ANOVA) followed by *post-hoc* Fisher’s PLSD test or Mann–Whitney *U*-test. A *p* value less than 0.05 denoted the presence of a statistically significant difference.

## Figures and Tables

**Table 1:** Altering mRNA expression of anti-oxidant-related factors or phase II, III detoxifying molecules in astrocytes treated with 6-OHDA on cDNA microarray.

	Mesencephalon Log_2_ Ratio	Control	6-OHDA	Striatum Log_2_ Ratio	Control	6-OHDA
GST (glutathione S-transferase A3)	1.87	1542.9	5646.9	5.30	315.4	12,397.4
GST (glutathione S-transferase Yc2 subunit (Gsta5))	1.61	718.6	2192.1	4.94	142.0	4359.8
Gpx (glutathione peroxidase 2)	1.31	22.0	54.5	1.10	33.2	71.1
NQO1 (NAD(P)H dehydrogenase)	2.48	1631.3	9083.3	3.46	1501.1	16,562.9
HO-1 (heme oxygenase-1)	0.78	2746.2	4718.5	3.41	2753.0	29,313.3
Catalase	0.64	317.3	493.7	1.13	213.3	466.7
G6PD (glucose-6-phosphate dehydrogenase)	0.35	11,557.4	14,765.4	1.83	8347.5	29,683.0
xCT (Xc-, Slc7a11)	−0.33	975.2	774.0	3.40	227.7	2398.7
MDR1 (p-glycoprotein, Abcb1)	0.76	7064.8	12,045.6	1.30	3693.6	9082.9
MRP4 (multidrug resistance-associated protein 4, Abcc4)	0.43	721.5	930.4	1.43	884.6	2335.4

Cultured mesencephalic or striatal astrocytes were treated with 6-OHDA (100 µM) for 24 h. The mRNA gene expression ratio was expressed in fluorescence intensity of 6-OHDA-treated group/fluorescence intensity of control group on a cDNA microarray. Log_2_ ratio = Log_2_ (signal intensity in the 6-OHDA-treated/signal intensity in the control). The increasing gene: Log_2_ ratio >1 if ratio was more than double, and the decreasing gene: Log_2_ ratio <−1 if ratio was less than one-half.
